# *Escherichia coli* isolates from vegetable farms in Addis Ababa, Ethiopia: Antimicrobial susceptibility profile and associated resistance genetic markers

**DOI:** 10.1002/fsn3.4071

**Published:** 2024-03-10

**Authors:** Woinshet Hailu, Haile Alemayehu, Lulit Hailu, Girmay Medhin, Gireesh Rajashekara, Wondwossen A. Gebreyes, Tadesse Eguale

**Affiliations:** ^1^ Aklilu Lemma Institute of Pathobiology Addis Ababa University Addis Ababa Ethiopia; ^2^ College of Health Sciences Addis Ababa University Addis Ababa Ethiopia; ^3^ Ethiopian Public Health Institute Addis Ababa Ethiopia; ^4^ Global One Health initiative (GOHi) The Ohio State University Columbus Ohio USA; ^5^ Department of Animal Sciences, College of Food, Agricultural, and Environmental Sciences The Ohio State University Wooster Ohio USA; ^6^ Department of Preventive Veterinary Medicine, College of Veterinary Medicine The Ohio State University Columbus Ohio USA; ^7^ Ohio State Global One Health Addis Ababa Ethiopia

**Keywords:** animal manure, antimicrobial resistance, *E. coli*, foodborne pathogens, resistance genes

## Abstract

The use of animal manure to fertilize soil is an emerging concern contributing to the transfer of antimicrobial‐resistant pathogens to vegetables. Hence, assessing antimicrobial susceptibility profile of *Escherichia coli* in vegetable farms is essential to design appropriate interventions against antimicrobial resistance (AMR) in the food chain. This study assessed antimicrobial resistance profile and associated genetic markers among *E. coli* isolated from vegetable farms fertilized with animal manure in Addis Ababa, Ethiopia. A total of 1044 samples were collected using convenience sampling: soil (*n* = 271), manure (*n* = 375), and vegetables (*n* = 398) from 81 vegetable farms in Addis Ababa, Ethiopia. Antimicrobial susceptibility test was conducted for 100 *E. coli* isolates and antimicrobial resistance genes (ARGs) were tested by polymerase chain reaction (PCR). Of the 1044 collected samples, 25.3% were positive for *E. coli*, with significantly higher prevalence in the manure sample and samples collected from Akaki Kality sub‐city (*p* < .05). The highest resistance rate was recorded for tetracycline (72%), followed by streptomycin (63%), and sulfamethoxazole +trimethoprim (56%). Multidrug resistance was detected in 61% of the *E. coli* isolates. The *aac(3)‐IV* (76.9%), *bla*_*TEM*_ (65.4%), *aadA* (*60.3%*), *tet(A)* (58.3%), and *sulI* (51.7%) were the commonly detected resistance genes. The current study showed a high burden of antimicrobial resistance among *E. coli* isolated from manure‐amended vegetable farms, with potential of playing a significant role in the dissemination of antimicrobial resistance in the food chain. Efforts should be made to reduce the burden of resistant organisms and ARGs through prudent use of antimicrobials in livestock and application of appropriate composting techniques before using manure as fertilizer.

## Graphical abstract

This study investigated the prevalence and antimicrobial susceptibility of *Escherichia coli* among vegetable farms in Addis Ababa, Ethiopia. The prevalence of *E. coli* was 32.3% in manure, 23.6% in soil, and 19.8% in vegetables. Multidrug resistance was detected in 61% of the *E. coli* isolates, with the highest resistance to tetracycline (72%), streptomycin (63%), and sulfamethoxazole+ trimethoprim (56%). *aac(3)‐IV* (76.9%), *bla*_*TEM*_ (65.4%), *aadA* (60.3%), *tet(A)* (58.3%), and *sulI* (51.7%) were the commonly detected antimicrobial resistance genetic markers.

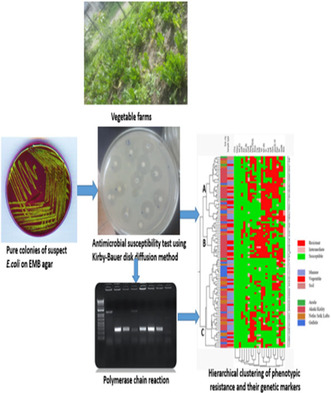

## 1 INTRODUCTION

Antimicrobial resistance (AMR) is designated by the United Nations as a public health crisis (IAGC, 2019) that complicates the treatment of infections and is known to increase morbidity and mortality (Smith & Coast, 2013). Antimicrobial resistance results in about 700,000 deaths worldwide. If no action is taken to reduce the burden of antimicrobial resistance, by 2050, the estimated number of deaths will reach up to 10 million, resulting in a cumulative economic loss of more than $100 trillion (O'Neill, 2016).

The emergence of antimicrobial‐resistant gram‐negative bacteria to the last‐line antimicrobials, such as carbapenems and colistin, indicates the severity of the problem (Liu et al., 2016). Since the late 1990s, the development of new antimicrobials has slowed dramatically, which has increased the necessity of preserving the efficacy of the current antimicrobials (Buchy et al., 2020). Even if the role of antimicrobial use in the development of antimicrobial resistance is well studied, the distribution and transmission of AMR is complex and poorly understood (Woolhouse et al., 2015). Host, bacterial, and environmental factors, exposure to antimicrobials in the animal production system, and poor waste disposal methods could contribute to the transmission of AMR (Holmes et al., 2016).

*Escherichia coli*, a member of the bacterial family *Enterobacteriaceae*, is one of the most widespread bacterial species globally. It is a commensal organism in the intestinal tract of animals and humans. However, various strains of *E. coli* are responsible for a broad spectrum of diseases in humans and animals (Ramos et al., 2020). Antimicrobial‐resistant *E. coli* is the most frequent cause of hospital‐ and community‐acquired infections (Pitout, 2012). Due to its capacity to carry different plasmids, *E. coli* is a major contributor to the horizontal transmission of drug resistance markers to other microorganisms (Salyers et al., 2004).

Multidrug‐resistant *E. coli* strains are responsible for transmitting antimicrobial resistance to the environment through several transmission pathways (Galindo‐Méndez, 2020). Thus, the environment plays a critical role by serving as a reservoir for the transmission of antimicrobial resistance genes (ARGs) (González Zorn & Escudero, 2012). *E. coli* serves as an indicator organism to monitor antimicrobial resistance, as it has a wide range of hosts (Bakshi et al., 2023; Fang et al., 2019).

There are a few studies conducted to identify *E. coli* strains in different food items and different farm environments in Ethiopia to understand its epidemiology, prevalence, and resistance profile (Dejene et al., 2022; Gemeda et al., 2023). However, the prevalence and antimicrobial resistance profile of *E. coli* from vegetable farms that apply dairy and poultry manure to their farms as fertilizer have not been well studied. Understanding this is essential to develop effective strategies to reduce the spread of AMR. Thus, this study aims to assess the spread of AMR in *E. coli* isolates from vegetables, manure, and soil from vegetable farms in Addis Ababa, Ethiopia.

## 2 MATERIALS AND METHODS

### 2.1 Study design, study area, and sample collection

A cross‐sectional study was conducted in Addis Ababa, the capital city of Ethiopia, from February 2022 to March 2023. Vegetable farms that use animal manure as fertilizer were selected from four sub‐cities of Addis Ababa (Akaki‐Kality, Nifas‐Silk Lafto, Arada, and Gulelle). Based on the availability of different numbers of farms in each sub‐city, 81 vegetable farms were visited: 41 from the Akaki Kality sub‐city, 23 from Nifas‐Silk Lafto, 14 from the Gulelle sub‐city, and 3 from the Arada sub‐city. Of the vegetable farms, 75.3% used composted manure, while the remaining 24.6% reported that they used animal manure without any treatment. Most of the vegetable farms, 95% (77 of 81), used dairy manure to fertilize their farms, while only 5% (4 of 81) used poultry litter.

From these farms, 1044 samples were collected: Akaki‐Kality (*n* = 482), Nifas‐Silk Lafto (*n* = 252), Arada (*n* = 72), and Gulelle (*n* = 238). Based on the sample type, 375 manure, 271 soil, and 398 vegetable samples were collected.

### 2.2 *Escherichia coli* isolation

*Escherichia coli* was cultured, as per the method described in ISO 16654:2001 (Mritunjay & Kumar, 2017). Briefly, 10 grams of the sample was suspended in 90 mL of buffered peptone water (BPW) and mixed by shaking to form a slurry. The slurry was incubated at 37°C for 24 hours. Enrichment and isolation of presumptive *E. coli* colonies and the biochemical test were performed, as described previously (Quinn et al., 2011). For identification of *E. coli* O157:H7, first, the *E. coli* isolates were inoculated on a sorbitol MacConkey agar plate, and all non‐sorbitol fermenter *E. coli* isolates were further tested using the PROLEX *E. coli* O157 latex test reagent kit as per the manufacturer's instruction.

### 2.3 Antimicrobial susceptibility testing

To represent the 264 *E. coli* isolates, one hundred *E. coli* isolates were selected proportionally from all the sample types (vegetables *n* = 30, dairy cattle manure *n* = 46, and soil *n* = 24) for the antimicrobial susceptibility testing. Antimicrobial susceptibility testing of the *E. coli* isolates was performed, according to the Clinical and Laboratory Standards Institute (CLSI) guidelines using the Kirby–Bauer disk diffusion method on Muller–Hinton agar plates (Oxoid, CM0337, Basingstoke, England) (CLSI, 2022). Antimicrobials used in the current study were: ampicillin (10 μg), nalidixic acid (30 μg), sulfamethoxazole+ trimethoprim (1.25/23.75 μg), sulfisoxazole (1000 μg), chloramphenicol (30 μg), ceftriaxone (30 μg), amoxicillin+clavulanic acid (20/10 μg), streptomycin (10 μg), kanamycin (30 μg), ciprofloxacin (5 μg), tetracycline (30 μg), gentamicin (10 μg), amikacin (30 μg), azithromycin (15 μg), and meropenem (10 μg). Antimicrobial disks used in this study were all from Sensi‐Disc, Becton Dickinson & Company, Loveton, USA. *E. coli* isolates were considered multidrug‐resistant (MDR) when they were resistant to at least one agent in three or more antimicrobial classes (Rafailidis & Kofteridis, 2022). *E. coli* ATCC 25922 was used for quality control when conducting antimicrobial susceptibility tests.

### 2.4 Detection of antimicrobial resistance genes

Bacterial DNA was extracted using the boiling method, as described previously (Islam et al., 2016). *E. coli* isolates that were phenotypically resistant to tetracycline, ampicillin, gentamicin, streptomycin, and sulfonamide were screened for eight antibiotic resistance genes: tetracycline‐resistant genes (*tet(A)*, *tet(B)*, and *tet(C)*), aminoglycoside‐resistant genes aminoglycoside acetyltransferase (*aac (3)‐IV*), and adenylyl transferase gene (*aadA*), sulfonamide‐resistant genes (*sulI* and *sulII*), and β‐lactamase gene (*bla*_*TEM*_), using conventional polymerase chain reaction (PCR). These ARGs were selected, based on the prevalence of phenotypic resistance to antimicrobials tested in this study. The PCR conditions and primer sequences used for this assay are shown in Table 1. The PCR product was observed using agarose gel electrophoresis.

**TABLE 1:** The sequence of primers used for the detection of antimicrobial resistance genes in *E. coli* isolates.

Antimicrobial	Resistance gene	Primer sequence 5′–3′	Annealing temp (°C)	Length (bp)	References
Tetracycline	*tet(A)*	F: GCTACATCCTGCTTGCCTTC R: CATAGATCGCCGTGAAGAGG	50	210	Bryan et al. (2004)
	*tet(B)*	F: TTGGTTAGGGGCAAGTTTTG R: GTAATGGGCCAATAACACCG	60	659	Ng et al. (2001)
	*tet(C)*	F: CTTGAGAGCCTTCAACCCAG R: ATGGTCGTCATCTACCTGCC	60	418	Ng et al. (2001)
Ampicillin	*bla*_*TEM*_	F: TTTCGTGTCGCCCTTATTCC R: ATCGTTGTCAGAAGTAAGTTGG	60	403	Mohammed et al. (2016)
Gentamicin	*aac(3)‐IV*	F: CTTCAGGATGGCAAGTTGGT R: TCATCTCGTTCTCCGCTCAT	55	286	Van et al. (2008)
Streptomycin	*aadA*	F: TATCCAGCTAAGCGCGAACT R: ATTTGCCGACTACCTTGGTC	55	447	Van et al. (2008)
Sulfonamides	*SulI*	F: TTCGGCATTCTGAATCTCAC R: ATGATCTAACCCTCGGTCTC	55	822	Van et al. (2008)
	*SulII*	F: CGGCATCGTCAACATAACCT R: TGTGCGGATGAAGTCAGCTC	50	721	Srinivasan et al. (2005)

*Note*: F, forward primer; R, reverse primer.

### 2.5 Statistical analysis

Descriptive statistical methods were used to summarize the number of samples collected and their prevalence. The chi‐square test was used to assess the association of different antimicrobial resistance patterns with the source of *E. coli* isolates. Statistical Product and Service Solutions (SPSS) version 26 was used to perform the descriptive analysis. Principal component analysis (PCA) and hierarchal clustering were used to investigate the association between the antimicrobial resistance pattern and the source of the isolates. JMP Pro 17 was used to plot the heat map representation with a dendrogram and the PCA. A *p*‐value less than 0.05 was considered an indicator of a statistically significant association.

## 3 RESULTS

### 3.1 Prevalence and distribution of *E. coli* isolates

From the 1044 collected samples, 264 (25.3%) were positive for *E. coli*. None of the *E. coli* isolates were *E. coli* O157:H7. The prevalence of *E. coli* in the manure sample (32.3%;121 of 375) and in those samples collected from Akaki Kality sub‐city (32.2%;155 of 482) was significantly higher compared to the prevalence of *E. coli* collected from other samples and those from other sub‐cities, respectively (*p* < .05). Moreover, the prevalence of *E. coli* was 23.6% (64 of 271) and 19.8% (79 of 398) in soil and vegetable samples, respectively. A similar 20% prevalence of *E. coli* was observed in Gulelle (48 of 238) and Nifas‐Silk Lafto (52 of 252) sub‐cities, whereas the lowest prevalence was detected in Arada sub‐city at 12.5% (9 of 72). The prevalence and distribution of *E. coli* isolated from samples collected in the four sub‐cities and from different sample types are shown in Table 2.

**TABLE 2:** Prevalence of *E. coli* from different sources of vegetable farms and different sub‐cities of Addis Ababa.

Characteristics	Category	No. of samples tested	No. (%) of positive samples	Chi square	*p*‐value
Sample type	Manure	375	121 (32.2)	16.29	.00028
	Soil	271	64 (23.6)		
	Vegetable	398	79 (19.8)		
Sub‐city	Arada	72	9 (12.5)	24.46	.00002
	Gulelle	238	48 (20.2)		
	Akaki‐Kality	482	155 (32.2)		
	Nifas‐Silk Lafto	252	52 (20.6)		
Total		1044	264 (25.28%)		

### 3.2 Antimicrobial susceptibility profile of *E. coli* isolates

**FIGURE 1:**
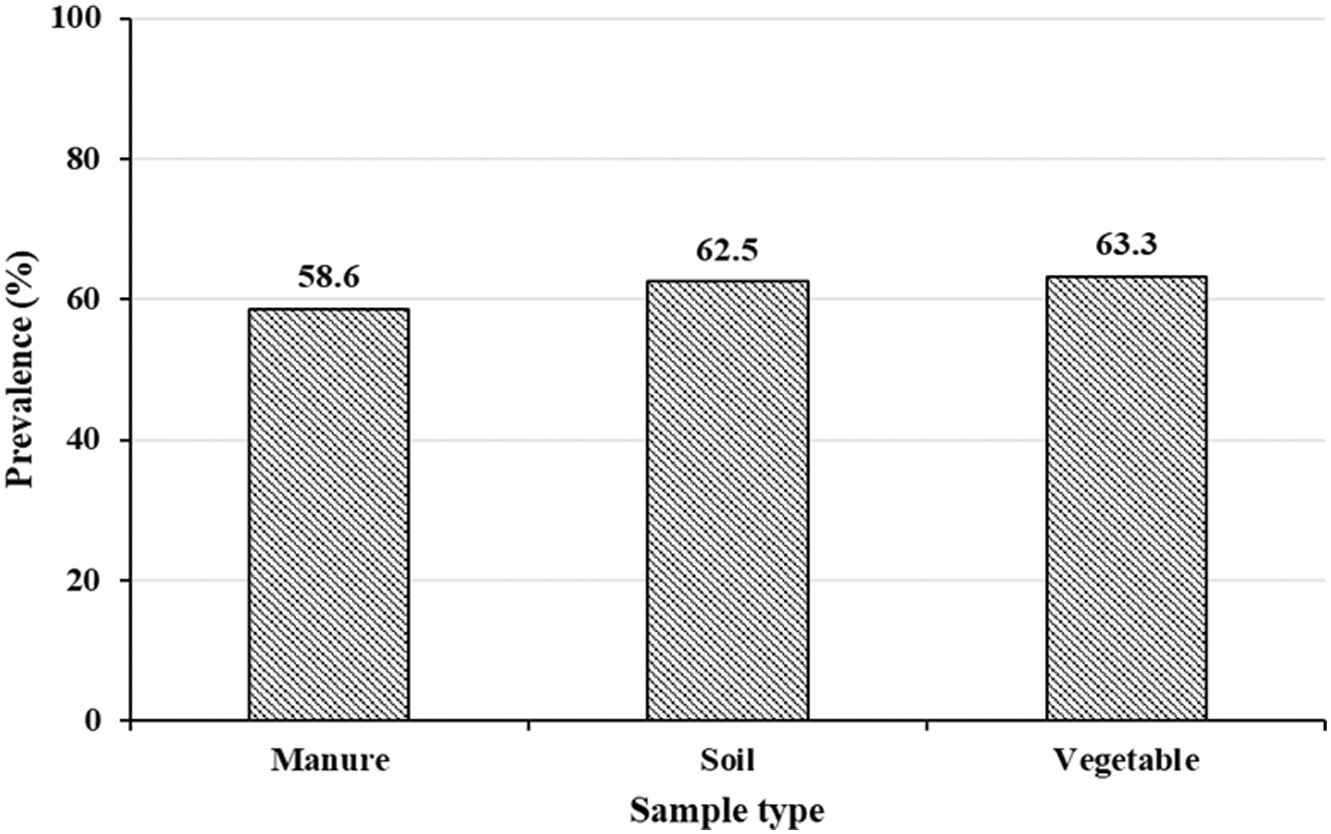
Rate of occurrence of multidrug‐resistant *E. coli* isolates from different sample types.

One hundred *E. coli* isolates were tested for their antimicrobial susceptibility. The highest resistance rate was detected for tetracycline (72%), followed by streptomycin (63%), and sulfamethoxazole+ trimethoprim (56%). Regardless of the source, all the *E. coli* isolates were susceptible to chloramphenicol and meropenem. Multidrug resistance was observed in 61% of the *E. coli* isolates, where 58.6%, 62.5%, and 63.3% of the *E. coli* isolates from manure, soil, and vegetables, respectively, were MDR (Figure 1). Among isolates from vegetables, 50%, 80%, 54.5%, and 83.3% of isolates from lettuce, cabbage, kale, and swiss chard, respectively, were MDR.

The highest rate of resistance to gentamicin was recorded for *E. coli* isolates from soil, 62.5% (15 of 24), followed by ampicillin, 58.3% (14 of 24), kanamycin, 45.8% (11 of 24), and sulfisoxazole, 58.3% (14 of 24). On the other hand, *E. coli* isolates from vegetables showed the highest rate of resistance to tetracycline: 76.6% (23 of 30), 30% (9 of 30) to nalidixic acid, and 23.3% (7 of 30) to ciprofloxacin. Resistance to amikacin was recorded in *E. coli* isolates from manure (8.7%), followed by 3.3% for isolates from vegetables, whereas all isolates from soil were susceptible (Table 3).

**TABLE 3:** Antimicrobial susceptibility profile of *E. coli* isolated from vegetable farms fertilized with animal manure.

Antimicrobials	No. of samples from different sources (% resistant)									Overall % R *n* = 100
	Manure (%) *n* = 46			Soil (%) *n* = 24			Vegetable (%) *n* = 30			
	R	I	S	R	I	S	R	I	S	R
Tetracycline	33 (71.7)	0	13 (28.3)	16 (66.6)	‐	8 (33.3)	23 (76.6)	‐	7 (23.3)	72
Chloramphenicol	0	0	46 (100)	0	0	24 (100)	0	0	30 (100)	0
Ampicillin	24 (51.2)	2 (4.3)	20 (43.4)	14 (58.3)	2 (8.3)	8 (33.3)	14 (46.6)	2 (6.6)	14 (46.6)	52
Gentamicin	22 (47.8)	7 (15.2)	17 (36.9)	15 (62.5)	3 (12.5)	6 (25)	15 (50)	2 (6.6)	13 (43.3)	52
Streptomycin	28 (60.6)	13 (28.2)	5 (10.8)	16 (66.6)	3 (12.5)	5 (20.8)	19 (63.3)	6 (20)	5 (16.6)	63
Kanamycin	17 (36.9)	11 (23.9)	18 (39.1)	11 (45.8)	2 (8.3)	11 (45.8)	12 (40)	3 (10)	15 (50)	40
Amikacin	4 (8.7)	9 (19.5)	33 (71.7)	0	8 (33.3)	16 (66.6)	1 (3.3)	4 (13.3)	25 (83.3)	5
Nalidixic acid	9 (19.5)	9 (19.5)	28 (60.6)	3 (12.5)	3 (12.5)	18 (75)	9 (30)	5 (16.6)	16 (53.3)	21
Ciprofloxacin	9 (19.5)	2 (4.3)	35 (76.0)	3 (12.5)	‐	21 (87.5)	7 (23.3)	2 (6.6)	21 (70)	19
Ceftriaxone	8 (17.4)	7 (15.2)	31 (67.4)	3 (12.5)	6 (25)	15 (62.5)	4 (13.3)	8 (26.6)	18 (60)	15
Amoxicillin+ Clavulanic acid	20 (43.5)	14 (30.4)	12 (26.0)	11 (45.8)	7 (29.1)	6 (25)	12 (40)	12 (40)	6 (20)	43
Sulfamethoxazole +Trimethoprim	25 (54.3)	‐	21 (45.6)	14 (58.3)	‐	10 (41.6)	17 (56.6)	‐	13 (43.3)	56
Sulfisoxazole	21 (45.6)		25 (54.3)	14 (58.3)	‐	10 (41.6)	16 (53.3)	‐	14 (46.6)	51
Azithromycin	3 (6.5)		43 (93.5)	1 (4.1)	‐	23 (95.8)	3 (10)	‐	27 (90)	7
Meropenem	0	0	46 (100)	0	0	24 (100)	0	0	30 (100)	0

Abbreviations: S, susceptible; I, intermediate; R, resistant.

**FIGURE 2:**
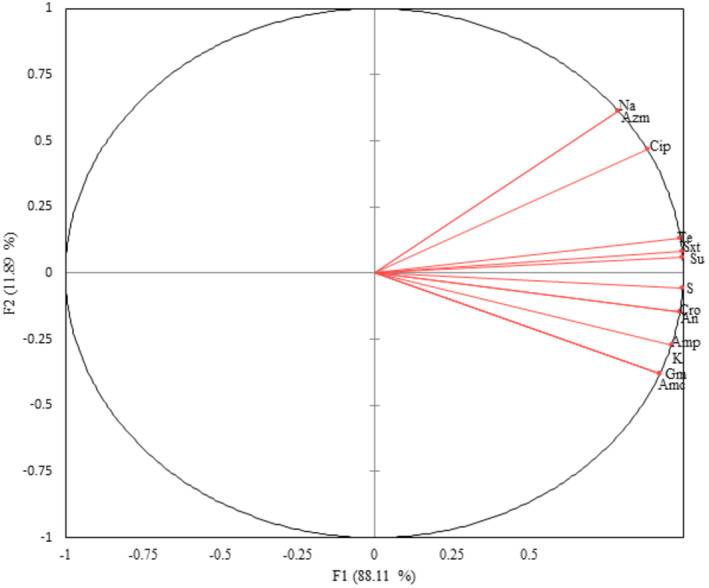
Principal component analysis of relative abundance of antimicrobial resistance among *E. coli* isolates. Amp: ampicillin, Na: nalidixic acid, Stx: sulfamethoxazole+ trimethoprim, Su: sulfisoxazole, Amc: amoxicillin+ clavulanic acid, S: streptomycin, K: kanamycin, Cip: ciprofloxacin, Te; tetracycline, Gm: gentamicin, An: amikacin, Cro: ceftriaxone, and Azm: azithromycin.

The principal component analysis grouped *E. coli* isolates that were resistant to sulfisoxazole, sulfamethoxazole+ trimethoprim, tetracycline, and streptomycin together, showing a similar trend of occurrence within these isolates. In addition, resistance to sulfisoxazole, sulfamethoxazole+ trimethoprim, tetracycline, and streptomycin among *E. coli* isolates was strongly correlated. However, *E. coli* isolates that were resistant to nalidixic acid, ciprofloxacin, and azithromycin stand separately, as they do not have a similar trend of resistance to other antibiotics (Figure 2).

### 3.3 Distribution of antimicrobial resistance genetic markers among *E. coli* isolates

Phenotypically resistant *E. coli* isolates were tested for their corresponding resistance genes. Resistance genes encoding for aminoglycoside resistance *aac(3)‐IV* were detected in 76.9% (40 of 52) of tested isolates, followed by *bla*_*TEM*_ detected in 65.4% (34 of 52), and *aadA* was detected in 60.3% (38 of 63) of isolates. On the other hand, *tet(A)*, *tet(B)*, and *tet(C)* were detected in 58.3% (42 of 72), 31.9% (23 of 72), and 4.2% (3 of 72) of tetracycline‐resistant isolates, respectively. Moreover, *sulI* and *sulII* were detected in 51.7 (29 of 56) and 41% (23 of 56) of sulfisoxazole‐resistant isolates, respectively. Based on sample type, the highest prevalence of the resistance genes was observed in isolates from soil: *aac(3)‐IV* (86.6%), *aadA* (81.2%), *bla*_*TEM*_ (71.4%), and *sulI* (57.1%). The detailed distribution of AMR genes in *E. coli* isolates from different sample sources is shown in Table 4.

**TABLE 4:** Prevalence of selected antimicrobial resistance genes in *E. coli* isolates obtained from manure, soil, and vegetables.

Encode resistance to	Source of *E. coli* isolates	No. of resistant isolates (%)	Tested resistant genes	No. of positive (%)	No. of *E. coli* isolates having common genes
Tetracycline	Manure	33	*tet (A)*	18 (54.5)	1 (*tet (A)* and *tet (B)*)
			*tet (B)*	13 (34.4)	
			*tet (C)*	0	
	Soil	16	*tet (A)*	8 (50)	2 (*tet (A)* and *tet (B)*)
			*tet (B)*	7 (43.7)	
			*tet (C)*	2 (12.5)	
	Vegetables	23	*tet (A)*	16 (69.5)	
			*tet (B)*	3 (13.0)	
			*tet (C)*	1 (4.3)	
Ampicillin	Manure	24	*bla*_*TEM*_	15 (62.5)	
	Soil	14	*bla*_*TEM*_	10 (71.4)	
	Vegetables	14	*bla*_*TEM*_	9 (64.2)	
Gentamicin	Manure	22	*aac(3)‐IV*	18 (81.8)	
	Soil	15	*aac(3)‐IV*	13 (86.6)	
	Vegetables	15	*aac(3)‐IV*	9 (60)	
Streptomycin	Manure	28	*aadA*	16 (57.1)	
	Soil	16	*aadA*	13 (81.2)	
	Vegetables	19	*aadA*	9 (47.3)	
Sulfonamide	Manure	25	*sulI*	13 (52)	
			*sulII*	10 (40)	
	Soil	14	*sulI*	8 (57.1)	1 (*sulI* and *sulII*)
			*sulII*	6 (42.8)	
	Vegetables	17	*sulI*	8 (47)	1 (*sulI* and *sulII*)
			*sulII*	7 (41.2)	

### 3.4 Antimicrobial resistance pattern of *E. coli* isolates and distribution of antimicrobial resistance genes

**FIGURE 3:**
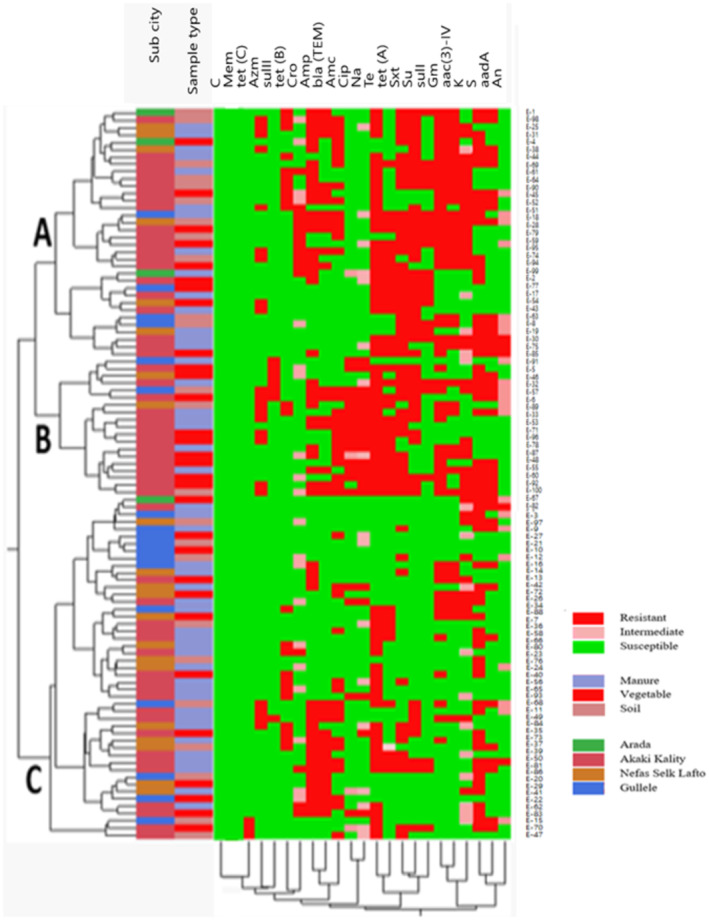
Hierarchical clustering of phenotypic resistance pattern and genetic markers of selected resistance phenotype of *E. coli* isolates. Amp: ampicillin, Na: nalidixic acid, Stx: sulfamethoxazole+ trimethoprim, Su: sulfisoxazole, Amc: amoxicillin+ clavulanic acid, S: streptomycin, K: kanamycin, Cip: ciprofloxacin, Te; tetracycline, Gm: gentamicin, An: amikacin, Cro: ceftriaxone, Mem: meropenem, and Azm: azithromycin.

The hierarchical clustering of *E. coli* isolates based on the phenotypic resistance pattern and distribution of ARGs is shown in Figure 3. *E. coli* isolates were grouped into three clusters, with Clusters A, B, and C having 34, 19, and 47 *E. coli* isolates, respectively. In Cluster A, most of the *E. coli* isolates that were resistant to sulfamethoxazole+ trimethoprim, sulfisoxazole, and tetracycline were grouped together. Moreover, *E. coli* isolates that were susceptible to azithromycin, ciprofloxacin, and nalidixic acid were grouped together, as also shown in the PCA. In this cluster, only three *E. coli* isolates were from the Arada sub‐city. Whereas in Cluster B, all the isolates were tetracycline‐resistant, and most quinolone‐resistant isolates were found in this cluster. This cluster is composed of 19 *E. coli* isolates, interestingly; 15 of the *E. coli* isolates were from the Akaki Kality sub‐city. The overall rate of occurrence of MDR is high among isolates in Clusters A and B and the majority of multidrug‐resistant *E. coli* isolates in these two clusters were from the Akaki Kality sub‐city (Figure 3). Cluster C contained a large number of *E. coli* isolates with less resistance to the tested antimicrobials. It is the only cluster that contained three *E. coli* isolates positive for *tet(C)* genes, where one isolate was from the Gulelle sub‐city and the other two from the Akaki Kality sub‐city.

## 4 DISCUSSION

*Escherichia coli* is considered a reliable indicator of food contamination (Disassa et al., 2017). The overall prevalence of *E. coli* in this study was 25.3%, which is lower than that reported in a study from Belgium, which was 37% (Holvoet et al., 2013) but higher than that mentioned in another study in Ethiopia, where the prevalence of *E. coli* in soil was 6.8% (Gemeda et al., 2023). The observed difference in the prevalence of *E. coli* could be the result of ecological and environmental factors. Soil samples in the current study were from vegetable farms fertilized by animal manure, which is believed to have a high load of *E. coli*. The length of time post application of manure and use of non‐composted and composted manure also affects the level of contamination of soil and vegetables by *E. coli* (Ingham et al., 2004).

The prevalence of *E. coli* in vegetable samples was 19.8% in this study, lower than that given in a study in south Ethiopia at 31.4% (Alemu et al., 2018) and in Addis Ababa, Ethiopia, 32.1% (Kechero et al., 2019), and higher than in China at 11% (Li et al., 2020). The observed difference in the prevalence of *E. coli* could be due to the difference in farming practices and the difference in the quality of water used for irrigation, which affects the pathogen level of the produce (Uyttendaele et al., 2015). In addition, the survival of *E. coli* on manure‐amended agricultural farms could be affected by the soil management system of the farm (Franz et al., 2005). In this study, no *E. coli* O157:H7 was detected, but previous studies reported *E. coli* O157:H7 in foods of animal origin in Ethiopia (Abebe et al., 2023; Hamid et al., 2018). The common source of contamination of food of animal origin with *E. coli* O157:H7 is the fresh feces of cattle (Ferens & Hovde, 2010). Manure and soil specimens in the current study might not be contaminated with fresh feces harboring *E. coli* O.157:H7.

Different studies showed that 30–90% of the antimicrobials administered to animals are excreted as the parent compound in their feces or urine. This could lead to contamination of agricultural soil with antimicrobials and their residues contributing to the selection of resistant organisms in the soil (Gros et al., 2019; Sarmah et al., 2006; Wei et al., 2019; Zhang, He, et al., 2019). In our study, we did not measure the concentration of antibiotics in the collected samples, but different studies showed that antimicrobials were detected in agricultural soil amended with animal manure (Gros et al., 2019; Srichamnong et al., 2021). Thus, soil amendment with animal manure can potentially increase the selection and spread of resistant bacteria (Litt et al., 2021). In this study, *E. coli* isolates from soil showed a high rate of resistance to ampicillin, gentamicin, kanamycin, and sulfisoxazole, compared to manure and vegetable samples. Thus, soil is an important reservoir of antimicrobial‐resistant bacteria and resistance genes (Nesme & Simonet, 2015).

Resistance to some of the tested antimicrobials in this study was lower than those mentioned in other studies in Ethiopia (Messele et al., 2017; Tadesse et al., 2018). For example, the observed resistance for tetracycline in this study was 72%, which was higher than other findings in Ethiopia: 46.4% (Sarba et al., 2019), 8% (Gemeda et al., 2023), and 47.6% (Messele et al., 2017). The difference in resistance of *E. coli* strains in these studies could be due to the difference in the use of antimicrobials in veterinary practices in different study areas. Tetracycline and oxytetracycline are widely used in dairy and poultry farms in urban and peri‐urban areas of Addis Ababa, where animal wastes contaminated by these drugs and antimicrobial‐resistant organisms are discharged to the environment, leading to contamination of farms by resistant organisms (Eguale, 2018; Eguale et al., 2016).

Amikacin is not used to treat or prevent infections in livestock in Ethiopia. However, recently, it has been used in the treatment of multidrug‐resistant infections in humans at some hospitals in Addis Ababa. In this study, resistance to amikacin was observed among a few *E. coli* isolates from manure and vegetables from the Akaki Kality sub‐city. This might be due to the contamination of Akaki River, with wastewater generated from hospital facilities in Addis Ababa, leading to contamination of manure and vegetables. Akaki River is the main source of water for the farms in the area (Yitayew et al., 2022).

A recent meta‐analysis study in Ethiopia showed that *E. coli* strains from foods, food handlers, animals, and the environment had high MDR profile next to *Staphylococcus* and *Salmonella* (Gemeda et al., 2021). In the current study, MDR was observed in 61% of the *E. coli* isolates, which is higher than 11.8% (Gemeda et al., 2023) and lower than 78.1% (Sarba et al., 2019) in Ethiopia. This variation could be due to the difference in geographical location of the study areas and variation in the number and types of antimicrobials used in animals. Interestingly, similar levels of MDR were reported in a study from South Africa: 61.5% (Fatoba et al., 2022) and 60.5% (Msimango et al., 2023). From the four sub‐cities, more MDR *E. coli* isolates were obtained from the Akaki Kality sub‐city. This could be due to the high contamination rate of the Akaki River by waste from health facilities, farms, and other human activities upstream (Tesfaye et al., 2019).

In this study, *E. coli* isolates from soil have the highest percentage of ARGs (*aac (3)‐IV*, *aadA*, *sulI*, and *bla*_*TEM*_) compared to those *E. coli* isolates obtained from manure and vegetables. The possible explanation for this observation could be that soil from the farms in the current study area is contaminated with human and animal waste from upstream areas with a high human population and livestock. Soil is reported to be the largest environmental reservoir of ARGs, accounting for approximately 30% of known ARGs (Nesme et al., 2014). Moreover, soil‐derived ARGs are an important source of resistance genes found in vegetables (Chen et al., 2017).

The prevalence of *tet(A)* (58.3%) and *sulI* (51.7%) was lower than those reported in another study in Ethiopia, *tet(A)* (65.1%*)* and *sulI* (54%) (Messele et al., 2017), and another study in Egypt, where *tet(A)* (64.2%) and *sulI* (56.7%) were reported (Ramadan et al., 2020). The prevalence of *tet(A)* and *bla*_*TEM*_ in this study was higher than that reported in a study from Tanzania, where the prevalence for both *tet (A)* and *bla*_*TEM*_ was 46% (Sonola et al., 2022). This difference might be attributed to difference in the type of manure amendment, the availability of different soil microbiota and the physicochemical characteristics of the soil, and variations in the distribution of genes encoding for resistance to antimicrobials in different regions (Montealegre Maria et al., 2018; Zhang, Hu, et al., 2019).

In this study, *tet(A)* was more common than *tet(B)*, showing the dominant role of *tet(A)* in conferring resistance to tetracycline in *E. coli* isolates circulating in the study area (Aworh et al., 2021). Additionally, some tetracycline‐resistant *E. coli* isolates in this study were negative for both *tet(A)* and *tet(B)*. One possible reason for the observed tetracycline resistance in these *E. coli* isolates could be due to other *tet* genes like *tet(O)* and *tet(M)* (Gao et al., 2012). Similarly, in *E. coli* isolates resistant to sulfisoxazole and negative to *sulI* and *sulII* genes, *sulIII* could be the gene encoding for the observed phenotypic resistance (Na et al., 2014). These resistance genes are mainly plasmid‐mediated and could be sources of resistance transferred from bacteria in manure to grown vegetables (Zhang, Hu, et al., 2019).

## 5 CONCLUSION

Recycling animal manure into agricultural soil is an emerging concern because it can transfer multidrug‐resistant foodborne pathogens to the produce. Our study showed that *E. coli* isolated from the agricultural environment and the produce were resistant to different antimicrobials and contained different resistant genes. This has a significant role in the dissemination of antimicrobial resistance in the food chain. Therefore, appropriate use of animal manure after proper composting is recommended to minimize the dissemination of foodborne pathogens and antimicrobial resistance in the food chain.

## AUTHOR CONTRIBUTIONS

**Woinshet Hailu:** Conceptualization (lead); data curation (lead); formal analysis (lead); investigation (lead); methodology (lead); software (lead); validation (lead); writing – original draft (lead); writing – review and editing (lead). **Haile Alemayehu:** Data curation (equal); formal analysis (equal); investigation (equal); methodology (equal); supervision (equal); validation (equal); writing – original draft (equal); writing – review and editing (equal). **Lulit Hailu:** Data curation (equal); formal analysis (equal); investigation (equal); methodology (equal); resources (equal); validation (equal); writing – review and editing (equal). **Girmay Medhin:** Conceptualization (equal); data curation (equal); formal analysis (equal); investigation (equal); methodology (equal); software (equal); supervision (equal); validation (equal); writing – original draft (equal); writing – review and editing (equal). **Gireesh Rajashekara:** Data curation (equal); formal analysis (equal); investigation (equal); methodology (equal); supervision (equal); validation (equal); writing – review and editing (equal). **Wondwossen Gebreyes:** Data curation (equal); formal analysis (equal); investigation (equal); methodology (equal); supervision (equal); validation (equal); writing – review and editing (equal). **Tadesse Eguale:** Conceptualization (lead); data curation (lead); formal analysis (lead); funding acquisition (lead); investigation (equal); methodology (equal); resources (lead); supervision (lead); validation (lead); writing – original draft (lead); writing – review and editing (equal).

## FUNDING INFORMATION

This study was supported by the Addis Ababa University thematic research project and Sustainable One Health Research Training Capacity (OHEART): Molecular epidemiology of zoonotic foodborne and waterborne pathogens in Eastern Africa, funded by the NIH Fogarty International Center (D43TW008650), through the Global One Health initiative (GOHi).

## CONFLICT OF INTEREST STATEMENT

The authors have no conflict of interest to declare.

## ETHICS STATEMENT

The Institutional Review Board of Aklilu Lemma Institute of Pathobiology reviewed the protocol for its ethical and methodological standards and approved the conduct of the study (Ref. No. ALIPB IRB/75/2014/22 on April 30, 2022).

## Data Availability

The authors confirm that all the data are included in the manuscript.
